# Relationship between diarrhoea risk and the combinations of drinking water sources in the Kathmandu Valley, Nepal

**DOI:** 10.1093/inthealth/ihab032

**Published:** 2021-06-11

**Authors:** Yuka Kobayashi, Yuri Ito, Sadhana Shrestha, Hiroshi Yokomichi, Kei Nishida

**Affiliations:** Special Graduates Program on River Basin Environmental Science, University of Yamanashi, 4-3-11 Takeda, Kofu, Yamanashi 400-8511, Japan; Special Graduates Program on River Basin Environmental Science, University of Yamanashi, 4-3-11 Takeda, Kofu, Yamanashi 400-8511, Japan; United Nations University-Institute for the Advanced Study of Sustainability, 5-53-70 Jingumae, Shibuya, Tokyo 150-8925, Japan; Institute for Future Initiatives (IFI), University of Tokyo, 7-3-1 Hongo, Bunkyo, Tokyo 113-8654, Japan; Faculty of Medicine, University of Yamanashi, 1110 Shimokato, Chuo, Yamanashi 409-3898, Japan; Interdisciplinary Centre for River Basin Environment, University of Yamanashi, 4-3-11 Takeda, Kofu, Yamanashi 400-8511, Japan

**Keywords:** alternative water sources, diarrhoea risk, jar water, questionnaire survey, tanker water

## Abstract

In Nepal, the number of diarrhoea hospitalizations in all ages is seriously high. According to the World Health Organization, diarrheal diseases can be substantially prevented through safe drinking water sources. In the Kathmandu Valley, because of the shortage of piped water, local residents use alternative water sources, such as groundwater, jars and tanker water. However, these alternative water sources can be contaminated. This study aimed to clarify the relationship between diarrhoea risk and the combinations of drinking water sources. A survey using multiple questionnaires on diarrhoea occurrence, water sources and water treatment was conducted three time between 2015 and 2016. The odds ratios (ORs) of developing diarrhoea were significantly high for drinking jar (OR 6.1) and tanker water (OR 8.4) compared with not drinking. The combined drinking of jar and tanker water obtained the 1 log higher OR compared with drinking only piped water. Conversely, drinking groundwater had a low OR, implying that the residents refrained from drinking polluted groundwater. In conclusion, diarrhoea occurrence was related not only to the level of water contamination, but also to a behavioural factor, i.e. people's careful management of the choice of multiple water sources.

## Introduction

Waterborne disease caused by contaminated water consumption can affect numerous people in a short time.^1,2^ Nearly 1000 children die daily because of preventable diarrheal diseases related to water and sanitation.^3^ Diarrheal disease is the second leading cause of mortality in children <5 y of age, with approximately 525 000 deaths annually.^4^ According to the World Health Organization, diarrheal diseases can be significantly prevented through safe drinking water and adequate sanitation and hygiene.^4^ While Sustainable Development Goal 6 aims to ensure access to water and sanitation for all by 2030, the routine use of multiple water sources to meet household water needs is widely practiced in developing countries across Asia and Africa.^5,6^ Although occurrences of diarrhoea caused by single water sources have been reported, particularly for groundwater,^7,8^ there are few studies on the combination of water sources impacting on diarrhoea.

In Nepal, the incidence of diarrhoea in children <5 y of age was 400 per 1000 in 2016^9^ and 1193 died in 2017.^10^ In areas where 59% of drinking water samples were contaminated with *Escherichia coli*, the *E. coli* concentration was associated with an increased prevalence of child diarrhoea.^11^ Also, diarrhoea and gastroenteritis are major causes of hospitalization in all ages.^9^ Kathmandu Upatyaka Khanepani Limited (KUKL) supplies piped water for people living in the Kathmandu Valley, which is our current study area. The population of the KUKL service area is approximately 2.56 million and 90% of this population is served by KUKL. The total water demand is 370 million litres per day (MLD); however, the KUKL supply is only roughly 144 and 86 MLD in the wet and dry seasons, respectively.^12^ Since one-third of households have no access to piped water^13^ and there is a limited supply for those who have access, local residents drink water from various alternative sources, including groundwater, jars and tanker water. These water sources are chemically and microbiologically polluted and are suspected to relate with diarrhoea.^14–17^ The potential infection risk of diarrhoea from multiple water sources with different enteropathogenic *E. coli* concentrations is highest in households drinking groundwater from shallow wells, followed by those drinking tanker water.^18^ The odds ratio (OR) for diarrhoea occurrence for those who use both improved and alternative water sources was 1.8-fold higher than in those who used only improved water.^19^ However, the detailed combinations of multiple drinking water sources and their impact on the occurrence of diarrhoea remains unknown. Hence this study aimed to clarify the relationship between the risk of diarrhoea and the combinations of drinking water sources.

## Methods

### Study area

**Figure 1.**
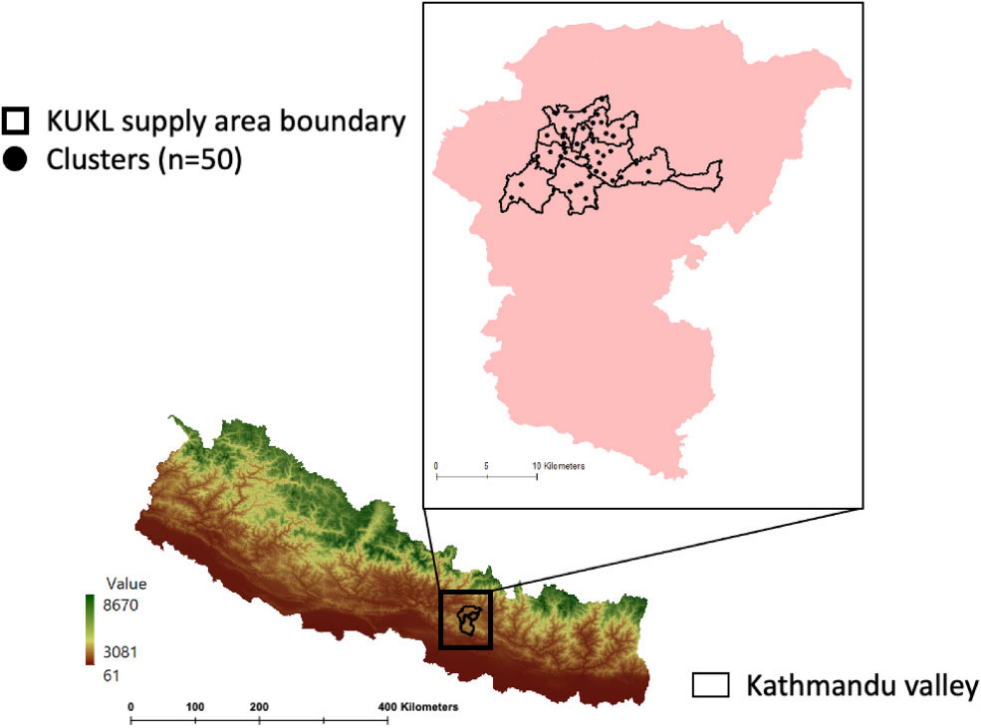
Map of Nepal showing the Kathmandu Valley and the distributions of survey clusters. The study area was divided according to the supply areas of the KUKL. Each cluster was randomly selected and 30 households closest to each selected geographical location were chosen.

Kathmandu is the capital city of Nepal, a low-income country in South Asia. The working age population (ages 15–59 y) is 64% and the gender distribution is 110 males per 100 females. The percentage of high school or college graduates is 58%. The average size of a household is 4.0. Piped water is the main source of drinking water for 62% of households. Groundwater is the main source of drinking water for 16%.^20^ The Kathmandu Valley is located in the central hilly region of Nepal between latitudes 27°32′13′′ and 27°49′10′′ north and longitudes 85°11′31′′ and 85°31′38′′ east (Figure 1). The valley is situated at 1300–1400 m above sea level and is drained by the Bagmati River and its tributaries. It has an area of 665 km^2^, covering 85% of the Kathmandu District, the entire Bhaktapur District and 50% of the Lalitpur District. Its population was 3.2 million in 2015, with a water demand of 370 MLD.^12^

### Questionnaire survey

This study used the results of a questionnaire survey conducted in densely populated areas of all KUKL water supply areas.^12^ The sample unit was a household and there are >40 000 households in our target area. We conducted two-stage cluster sampling. In the first stage, the probability of selecting a cluster was calculated using the household number in a ward, total number of households in the municipality or metropolitan or submetropolitan city and the total number of clusters to be sampled (i.e. 50). In the second stage, the probability of selection of a household in a ward was calculated using the total number of households in the ward and the number of households to be sampled (i.e. 30). Then weights were calculated using the probability of each cluster being sampled and the probability of each household being sampled in each selected cluster. As for the geographical location, 50 clusters were randomly selected using GIS, whereas the interviewers randomly selected 30 households closest to each selected cluster at the time of survey. They conducted face-to-face interviews with one of the household members between 15 and 60 y of age. Details of the questionnaires are presented elsewhere.^21^

A structured questionnaire survey was conducted three times between 2015 and 2016. Phase 1, the baseline period, was conducted during the dry season from January to April 2015, but only in 39 clusters (1139 households) due to the Gorkha earthquake. Phase 2 was conducted in 50 clusters (1500 households) during the dry season from December 2015 to February 2016. Phase 3 was conducted in 50 clusters (1500 households) during the wet season from August to September 2016. Hence we analysed all the households that participated in the three surveys. After excluding questionnaires that were answered by family members of invalid age or have missing answers, the data from 3903 households were included in this study. The style of questionnaire survey has an advantage in collecting information on persons both infected and uninfected or persons who did or did not go the hospital because of diarrhoea. Among the questionnaire items, diarrhoea incidence and drinking water sources were our main focus. In the present study, diarrhoea was identified by asking the following question: ‘Did you or your family experience diarrhoea (three or more loose or liquid stools per day) in the last 2 weeks?’. The water source for drinking was identified by asking ‘What is the purpose of using each water—Drinking: Yes vs. No?’.

### Statistical analysis

The use of drinking water sources and the onset of diarrhoea served as the explanatory and objective variables, respectively. Considering that the objective variable was binary, we analysed the OR by binary logistic regression analysis. The correlation of each variable was confirmed using the φ coefficient. Then the adjusted ORs were compared with the crude ORs. The variables for adjusting OR included four drinking water sources. The significance level was <0.05 for all analytical procedures. In addition, 95% confidence intervals were calculated. For all statistical analyses we used SPSS Statistics version 26.0 (IBM, Armonk, NY, USA).

## Results

### Descriptive statistics of diarrhoea and drinking water sources

After excluding households that did not answer about diarrhoea or drinking water sources, this study analysed 3903 households. Diarrhoea developed among family members from 38 households, with an incidence rate of 0.97%. We described 15 combinations of drinking water sources. Table 1 lists the number of households that used each drinking water combination, with and without diarrhoea. The households were also grouped into four categories: jar, piped, groundwater and tanker water sources. Diarrhoea developed in 2 of 910 households who only drank piped water, 10 of 1949 households who only drank jar water, 8 of 574 households who drank jar and piped water, 4 of 64 households who drank jar and tanker water and 12 of 104 households who drank jar, tanker and piped water. The combination of jar, tanker and piped water had the highest diarrhoea incidence. Also, two households who drank contaminated groundwater developed diarrhoea.

**Table 1.** Combinations of drinking water sources and the number of households with and without diarrhoea and households with family members who developed diarrhoea in the previous 2 weeks

		Households, n	
Group No.	Combination of drinking water sources	Diarrhoea yes	Diarrhoea no
1	① Piped	2	908
2	② Jar	10	1939
	③ Jar + piped	8	566
	④ Jar + groundwater	0	55
	⑤ Jar + piped + groundwater	0	13
3	⑥ Jar + tanker	4	60
	⑦ Jar + tanker + piped	12	92
	⑧ Jar + tanker + groundwater	0	5
	⑨ Jar + tanker + piped + ground	0	2
4	⑩ Groundwater	2	66
	⑪ Tanker	0	76
	⑫ Piped + groundwater	0	19
	⑬ Piped + tanker	0	62
	⑭ Groundwater + tanker	0	1
	⑮ Piped + groundwater + tanker	0	1
	Total	38	3865

### ORs for diarrhoea occurrence among family members according to the type of drinking water source

The φ coefficient indicates the correlation of each explanatory variable. Table 2 presents the φ coefficient for drinking or not drinking from each water source. Clearly, the φ coefficient of piped and jar water had the highest negative correlation (−0.57). However, the absolute values of the φ coefficient for other drinking water sources were all ≤0.12. Of note, many households also drank from a single drinking water source.

**Table 2.** φ coefficient relationship between households that drank each water source and those that did not.

Drinking water source	Piped	Groundwater	Jar	Tanker
Piped	1			
Groundwater	−0.09*	1		
Jar	−0.57*	−0.12*	1	
Tanker	0.06*	−0.02	−0.10*	1

*p<0.05.

Table 3 shows the ORs of diarrhoea occurrence among family members according to the type of drinking water source. The ORs were estimated separately for each water source comparing those who had diarrhoea with those who did not. Considering that various combinations of drinking water sources were included, the number of households with diarrhoea per the number of households that drank each water source did not add up to 38. According to the crude OR, diarrhoea most likely developed in households that drank tanker water (OR 8.7), followed by those that drank jar water (OR 3.5), piped water (OR 1.8) and groundwater (OR 1.3). However, the p-values of drinking piped and groundwater separately were insignificant. In households drinking jar water, the adjusted OR (6.1) was considerably higher than the crude OR (3.5). For other drinking water sources, the adjusted and crude ORs were insignificant.

**Table 3.** Binary logistic analysis results on the number of households drinking from each water source and the ORs for diarrhoea occurrence among family members according to the type of drinking water source.

Drinking water source	Households with diarrhoea/Households drinking each water source	Crude OR (95%CI)	p-Value	Adjusted OR (95% CI)	p-Value
Piped	22/1685	1.8 (0.95 to 3.5)	0.070	2.5 (1.2 to 5.0)	0.019
Groundwater	2/164	1.3 (0.30 to 5.3)	0.745	1.9 (0.44 to 8.3)	0.373
Jar	34/2766	3.5 (1.3 to 10)	0.017	6.1 (2.1 to 18)	0.001
Tanker	16/315	8.7 (4.5 to 17)	0.006	8.3 (4.2 to 17)	<0.001

Model adjusted for relevant variables including family size.

### ORs for diarrhoea occurrence among family members according to the type of drinking water source combinations

Table 4 presents the ORs for diarrhoea occurrence among family members according to the type of drinking water source combinations. Among the four drinking water sources, piped water was the cleanest. Piped water was also drunk in combination with other water sources, hence exclusive drinking of piped water was included in the reference category, which consisted of 920 households, of which 2 developed diarrhoea. The crude OR of the exclusive drinking of jar water or drinking jar water in combination with other water sources excluding tanker water was 3.2. Others obtained a crude OR of 4.0. Households that drank both jar and tanker water had the highest likelihood of diarrhoea (OR 46) and had a higher crude OR than those that drank other combinations of drinking water sources.

**Table 4.** ORs for diarrhoea occurrence among family members according to the type of drinking water source combinations

Drinking water source	Households, n	Having diarrhoea among family members	Crude OR (95% CI)	p-Value
Drinking only piped	920	2	1	
Drinking jar^a^	2591	18	3.2 (0.74 to 14)	0.122
Drinking jar and tanker^b^	175	16	46 (10 to 200)	<0.001
Others	227	2	4.0 (0.57 to 29)	0.164

a Drinking jar water only or drinking jar water in combination with other water sources excluding tanker water.

b Drinking both jar and tanker water or jar, tanker and other water sources.

## Discussion

In this study, the relationship between the risk of diarrhoea and the selection of drinking water sources was clarified. We demonstrated the φ coefficient relationship between households that drank each water source and those that did not (Table 2). Drinking piped water had a strong negative correlation with drinking jar water, therefore households with an insufficient piped water supply are likely to rely on jar water. Furthermore, the ORs for diarrhoea occurrence among family members were determined according to the type of drinking water source. Drinking jar water had a significantly higher adjusted OR than drinking piped water (Table 3). Therefore, although jar water is commonly a substitute for piped water, jar water is not safe to drink compared with piped water because of its high risk of developing diarrhoea.

According to a previous research, there are no significant differences in ORs of developing diarrhoea between households that drink improved and unimproved water sources in Nepal.^22^ However, given that many households use more than one water source in this study area, we should focus on the combinations of drinking water sources. Few households drank only piped water, which was the safest drinking water source, while many households drank piped water combined with other drinking water sources. In this study we found the ORs for diarrhoea occurrence among family members according to the type of drinking water source combinations (Table 4). The crude OR for combined jar and tanker water drinking was significantly high. This finding does not mean that drinking only jar water is associated with a higher risk of diarrhoea; rather, drinking combined jar and tanker water was associated with a higher risk of diarrhoea.

Moreover, 92% of the jar water samples and 77% of the tanker water samples collected in the Kathmandu Valley exceed the total coliform count limits defined by the Nepal Drinking Water Quality Standards.^16,17^ In the same region, *E. coli* was detected in 52% of the filling station samples and more frequently in tanker samples.^23^ Therefore the selection of drinking water sources requires careful management.

We recommend several measures to acquire safe drinking water. Considering that jar water^24^ and tanker water^25^ are sold by private companies, the government must intervene and inspect water quality. Treatment of drinking water in each household is also important for diarrhoea prevention, and jar and tanker water sources should also be treated. In the urban areas of the Kathmandu Valley, 75% of the households used single or multiple water treatment methods to purify their drinking water.^26^ The proportion of pretreatment of drinking water should also be increased. In other countries, local residents drink water from multiple sources. In downtown Kampala, Uganda, kaveras (drinking water sold in plastic bags) and jerry cans are biologically contaminated.^6^ Hence the relationship between diarrhoea and the combinations of drinking water sources may be examined in detail in other countries.

This study has some limitations. First, due to the small number of diarrhoea cases, the 95% CIs of the ORs were relatively wide. However, we believe that comparisons of the risk between groups have been preserved. This study was conducted during the rainy and dry seasons of 2015 and 2016. Childhood diarrhoea is reportedly significantly associated with an increase in the maximum temperature and rainfall in the Kathmandu Valley.^27^ Also, this study area is divided into nine water supply areas.^18^ Also, given that the age of the person with diarrhoea was not determined, we do not know whether the person with diarrhoea was <5 y old.

## Conclusions

Local residents in the Kathmandu Valley generally use multiple water sources for drinking. Our study showed that the ORs were significantly high for drinking jar and tanker water separately and the combination of these two water sources obtained the highest OR. Conversely, drinking groundwater had a low OR, suggesting that the residents refrained from drinking polluted groundwater. The selection of drinking water sources was related to careful management. Therefore managing the water quality of jar and tanker water sources and increasing the proportion of pretreatment of drinking water are necessary.

## Data Availability

Data are available from the authors upon reasonable request.
